# Benzobisthiadiazole and Its Derivative-Based Semiconducting Polymer Nanoparticles for Second Near-Infrared Photoacoustic Imaging

**DOI:** 10.3389/fchem.2022.842712

**Published:** 2022-02-24

**Authors:** Xuelong Huang, Ning Lan, Yanfeng Zhang, Wei Zeng, Haifeng He, Xiuhong Liu

**Affiliations:** ^1^ Key Laboratory of Prevention and Treatment of Cardiovascular and Cerebrovascular Diseases, Ministry of Education, Key Laboratory of Biomaterials and Biofabrication in Tissue Engineering of Jiangxi Province, College of Medical Information Engineering, Gannan Medical University, Ganzhou, China; ^2^ Jiangxi Engineering Laboratory of Waterborne Coating, School of Chemistry and Chemical Engineering, Jiangxi Science and Technology Normal University, Nanchang, China

**Keywords:** photoacoustic imaging, near-infrared-II, semiconducting polymer, nanoparticles, benzobisthiadiazole, thiadiazoloquinoxaline, thiadiazolobenzotriazole

## Abstract

Photoacoustic (PA) imaging has received more and more attention on disease diagnosis and fundamental scientific research. It is still challenging to amplify their imaging ability and reduce the toxicity of inorganic materials and exogenous contrast agents. Semiconducting polymer nanoparticles (SPNs), as a new type of contrast agent, have the advantages of low toxicity, flexible structure adjustment, good photostability, and excellent photothermal conversion efficiency. SPNs containing benzo(1,2-*c*;4,5-*c*′)bis(1,2,5)thiadiazole (BBT) units, as the most classic second near-infrared window (NIR-II, 1,000–1700 nm) PA contrast agents, can achieve light absorption in the NIR-II region, thereby effectively reducing light loss in biological tissues and improving imaging resolution. This mini review summarizes the recent advances in the design strategy of BBT and its derivative-based semiconducting polymer nanoparticles for second near-infrared photoacoustic imaging. The evolution process of BBT blocks provides a unique perspective for the design of high-performance NIR-II PA contrast agents.

## Introduction

Photoacoustic (PA) imaging is a hybrid imaging technology based on light excitation and ultrasound detection, which is widely used in monitoring surgery, visualization of blood vessels, and early detection of disease biomarkers (Zhen et al., 2021). The PA imaging process consists of three stages (Hong et al., 2017). First, a safe non-ionizing laser pulse is used to irradiate the corresponding biological tissue, and the photon energy is converted into heat in a short time. Second, the localized heat inside tissues undergoes transient thermoelastic expansion to generate ultrasonic waves. Finally, the generated ultrasonic signals are collected by a broadband ultrasonic transducer and converted into PA images. Therefore, PA imaging not only has the advantage of the sensitive light absorption in contrast to an optical method but also has the advantage of small acoustic scattering similar to an acoustic method, and exhibits better spatial resolution and imaging depth than traditional optical imaging (Yin et al., 2021).

The excitation light source is an important factor affecting PA imaging. Compared with ultraviolet and visible light, near-infrared light has relatively weaker interaction force in biological tissues, which is more conducive to clinical diagnosis (Lyu et al., 2019). Depending on the wavelength, the near-infrared range includes the first (NIR-I, 650–950 nm) and the second near-infrared wavelength ranges (NIR-II, 1,000–1700 nm). Due to obvious advantages in penetration depth and signal-to-noise ratio (SNR), PA imaging in the NIR-II window (1,000–1700 nm) has aroused increasing interest among clinicians and biomedical researchers. PA imaging contrast agents in the NIR-II window have a relatively weak extinction ability and can improve SNR by reducing background fluorescence and photoacoustic signals .

PA imaging contrast agents in the NIR-II window mainly focus on inorganic materials, including metallic nanoparticles, quantum dots, carbon materials, and rare-earth nanoparticles (Cheng et al., 2020). Although such kinds of inorganic agents have good performance in extinction coefficient and light stability, they cannot overcome the potential biological toxicity caused by heavy metal ions and metabolic problems. In contrast, organic material-based PA imaging contrast agents have good biocompatibility and can effectively avoid the toxicity of heavy metal ions to organisms (Jiang et al., 2019). Furthermore, the advantages of good photostability, light stability, and adjustable absorption properties indicate that organic materials, especially π-conjugated organic semiconducting polymers, are an excellent choice for NIR-II fluorescence imaging .

In recent decades, a large number of π-conjugated organic semiconducting polymers with an absorption range up to the near-infrared range have been developed, which can be used to improve power conversion efficiency (PCE) of polymer solar cells by capturing more photons in the NIR range. Benzo(1,2-*c*;4,5-*c*′]bis(1,2,5)thiadiazole (BBT), which consists of four electron-deficient C=N bonds, is considered to be the strongest electron-deficient (acceptor, A) unit (Karikomi et al., 1995). For instance, Reynolds et al. reported a donor–acceptor (D–A)-conjugated polymer P(DTP-BThBBT) by combining dithieno(3,2-*b*:2′,3′-*d*)pyrrole (DTP) as the D unit with BBT as the A unit, which shows maximum absorption at 1,231 nm (Steckler et al., 2009). It is indicated that D-A polymers consisting of a BBT unit are a promising class of semiconducting polymers (SPs) for the synthesis of efficient PA contrast agents used within the NIR-II biological window.

In this mini review, we summarize the recent progress of SPNs consisting of a BBT unit or its derivatives for NIR-II PA imaging. First, we discuss the chemical structures and design strategy of SPNs. The development trend of BBT-based SPNs and the perspectives are given subsequently.

## Molecular Designs

Molecular engineering of SPs plays a crucial role in PA imaging quality, including the absorption property, radiative decay rate (k_r_), SNR, and tissue penetration depth (Lei and Zhang, 2021). According to the constitution of repeating units, the conjugated backbones of the PA imaging contrast agents can be classified into two forms: a quinoid polymer and donor–acceptor (D–A) polymer (Huang et al., 2021). Empirical formula *E*_*g*_ = 1,240/λ shows that in order to achieve the NIR-II window SPNs, the bandgap of the SPs should be less than 1.24 eV. The process of converting the aromatic resonance into the quinone resonance of the quinone polymer is accompanied by the reduction of its bandgap. The bandgap of D–A polymers can be easily tuned by selecting D and A units of different electron-donating/withdrawing capabilities to produce the intramolecular charge transfer (ICT) effect. The D unit and the A unit are coupled through a palladium-catalyzed coupling reaction to obtain a D–A polymer. The extended conjugated backbone promotes the delocalization of electrons to reduce the bandgap. The perturbation theory explains that the narrow bandgap is formed by the hybridization of molecular orbitals after the polymerization of D and A units to produce a new higher-lying highest occupied molecular orbital (HOMO) and a new lower-lying lowest unoccupied molecular orbital (LUMO). Most of the reported NIR-II SPs are developed based on the D–A polymer strategy (Zhou et al., 2012).

**SCHEME 1:**
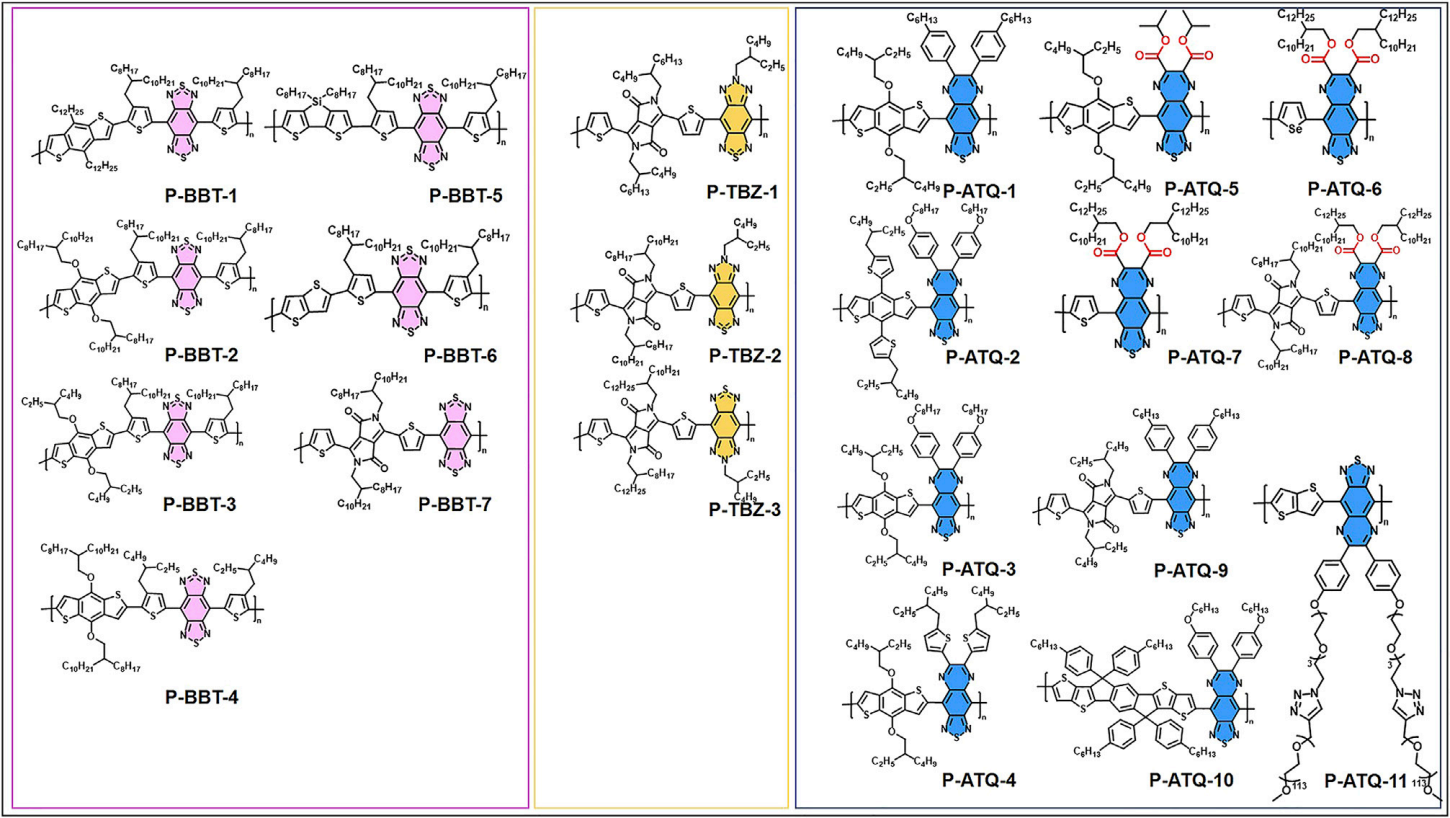
Chemical structures of BBT, TBZ, and ATQ-based SPs for NIR-II PA imaging.

Electronically, thiophene is a strong electron-rich unit. Due to the characteristics of thiophene, donors fusing with thiophene units are ideal donor choices for SPs, including typical donors such as cyclopentadithiophene (CDT), dithienosilole (DTS), and dithienopyrrole (DTP) (Zhou et al., 2013). On the other hand, BBT, a strong acceptor with an electron-deficient ability containing 4 C=N bonds, is widely used in the construction of SPNs for NIR-II PA imaging (Zhang et al., 2019). The D–A polymers based on the “strong donor–strong acceptor” strategy have been designed to result in NIR-II SPNs *via* a strong ICT, including many BBT-based polymers. However, the large planar structure of BBT, which fuses with two thiadiazole rings without solubilizing alkyl chains, reduces the solubility of BBT-based SPs in organic solvents. To overcome this challenge, the thiadiazole ring of BBT was replaced with a triazole or pyrazine ring to obtain (1,2,5)thiadiazolo(3,4-*f*)benzotriazole (TBZ) or thiadiazoloquinoxaline (ATQ) which can flexibly introduce alkyl side chains to improve polymer solubility (Dong et al., 2013; Luo et al., 2021). This account classifies NIR-II SPNs according to their strong acceptor units, which include BBT, TBZ, and ATQ (Scheme 1).

## Design of Near-Infrared Window-II Semiconducting Polymer Nanoparticles for Photoacoustic Imaging

### BBT-Based Near-Infrared Window-II Semiconducting Polymer Nanoparticles

Since Yamashita first synthesized a narrow bandgap polymer containing BBT units in 1995, BBT, as a classic strong electron-withdrawing unit building block, is widely used in organic semiconductor devices (Karikomi et al., 1995). Pu et al. chose different donor units including [4,8-bis((2-ethylhexyl)oxy] benzo(1,2-*b*:4,5-*b′*)dithiophene-2,6-diyl)bis(trimethylstannane), 2,6-bis(trimethyltin)-4,8-didodecylbenzo(1,2-*b*;4,5-*b′*)dithio-phene, and 2,5-bis(trimethylstannyl)thieno(3,2-*b*)thiophene copolymerized with BBT to form a series of degradable NIR-II SPNs (P-BBT-1, P-BBT-3, and P-BBT-6) (Jiang et al., 2019). The absorption of P-BBT-1 and P-BBT-2 is similar, and the absorption edge is at 1,200 nm. For increasing the electron-donating ability of the donor unit, the absorption edge is extended to 1,500 nm in P-BBT-6, whose backbone consists of thieno[3,2-*b*]thiophene and BBT units. P-BBT-1 is coprecipitated with PLGA-PEG to yield water-soluble nanoparticles, which shows a photothermal conversion efficiency (PTCE, *η*) of 53% under a 1,064-nm laser irradiation. The *η* of SPNs-(P-BBT-3) is 36%, followed by SPNs-(P-BBT-6) (49%). The particle size of SPNs of P-BBT-1 is degraded from 30 to 1 nm under the action of MPO and lipase, corresponding to the result that the complete metabolism of SPNs can be completed by the kidney and liver in 15 days in living mice. Furthermore, NIR-II PA imaging of P-BBT-1 exhibits a high signal-to-background ratio (SBR) of 4.6 and 2.3, respectively, in the tumor and brain vasculature model (Table 1). Liu et al. developed SPNs of P-BBT-2 for precise PA imaging and photothermal therapy (PPT) in the scalp and skull, which optimizes the side chain of BDT building block with 2-(octyldodecyl)oxy compared with P-BBT-3 (Guo et al., 2018). SPNs of P-BBT-2 decorated with cyclo[Arg-Gly-Asp-D-Phe-Lys(mpa)] show an SBR of up to 90 and an imaging depth of 3 mm in the scalp and skull, and it being combined with PPT can effectively extend the survival spans of brain tumor of mice. SPNs of P-BBT-4, which are appended to alkyl side chains 2-ethylhexyl (Eh) in the thiophene (T) units, would be used to investigate the relationship between the laser excitation wavelengths and the PA imaging ability, indicating that the SBR of 1,064 nm is better than other wavelengths (Guo et al., 2017). The SBR value of P-BBT-4 in brain tumor imaging is higher than that of other types of contrast agents, including MoS_2_ and perylene diimide.

**TABLE 1:** Summary of the properties and applications of representative NIR-II SPNs discussed in this review (λ_onset_, the onset of absorption value; λ_max_, the absorption peak value; Ex, excitation wavelength; *η*, the photothermal conversion efficiency; and NA, not applicable).

SPNs	λ_onset_ (nm)	λ_max_ (nm)	Ex (nm)	Properties	Disease model	References
P-BBT-1	1,320	1,079	1,064	SBR = 2.3	Brain	Jiang et al. (2019)
				*η* = 53%		
P-BBT-2	1,280	1,064	1,064	SBR = 90	Brain tumor	Guo et al. (2018)
P-BBT-3	1,230	1,079	1,064	*η* = 36%	Brain	Jiang et al. (2019)
P-BBT-4	1,280	1,064	1,064	SBR = 59	Orthotopic brain tumor	Guo et al. (2017)
P-BBT-5	1700	1,150	1,064	*η* = 65%	HepG-2 tumor cells	Wei et al. (2020)
P-BBT-6	1900	1,079	1,064	*η* = 49%	Brain	Jiang et al. (2019)
P-BBT-7	>1,500 nm	1,300	1,064	*η* = 60%	Breast tissue	Zhang et al. (2019)
P-TBZ-1	1,400	1,064	1,064	Imaging depth = 4 cm (breast tissue), 3.8 mm (skull)	Breast tissue/brain tumor	Yang et al. (2019)
P-TBZ-2	1,450	1,170	1,064	SBR = 22.3 dB; depth = 1,001 µm	Cerebral/tumor vasculatures	Guo et al. (2019)
P-TBZ-3	1,400	1,064	1,064	*η* = 53%, MPE = 0.5 W cm^−2^	Tumor-bearing mice	Men et al. (2020)
P-ATQ-1	1,200	929	1,064	*η* = 21.2%	Brain tumor	Wen et al. (2020)
P-ATQ-2	1,130	990	1,064	Contrast enhancement = 21.7-fold	Subcutaneous/brain	Yin et al. (2018)
P-ATQ-3	1,150	897	1,064	NA	Situ hepatic tumor	Zha et al. (2020)
P-ATQ-4	1,200	905	1,064	NA	Situ hepatic tumor	Zha et al. (2020)
P-ATQ-5	1,380	1,109	1,064	*η* = 61.6%	Situ hepatic tumor	Zha et al. (2020)
P-ATQ-6	1,550	1,140	1,064	NA	Brain vasculature	Luo et al. (2021)
P-ATQ-7	2000	1,270	1,064	Depth = 10 mm	Brain vasculature	Luo et al. (2021)
P-ATQ-8	2,214	1,500	1,064	NA	Brain vasculature	Luo et al. (2021)
P-ATQ-9	NA	1,253	1,064	Depth = 3 cm	Brain vasculature	Jiang et al. (2017)
P-ATQ-10	1,060	930	980	Small vessels = ∼2 μm	The whole body	Yang et al. (2020)
P-ATQ-11	1,350	1,000	1,064	*η* = 30.53%, imaging depth = 1.5 cm	Breast tissue	Yin et al. (2020)

Liu et al. synthesized P-BBT-5 by combining DTS as a donor unit with BBT as an acceptor unit, which has a stronger ICT effect than P-BBT-4 that makes the absorption peak of P-BBT-5 redshift to 1,150 nm (Wei et al., 2020). The *η* of SPNs P-BBT-5 is up to 65%, and the SPNs show excellent targeting capability in PA imaging of cancers. Diketopyrrolopyrrole (DPP) is a promising building block as a natural pigment to construct low bandgap polymers which exhibits strong electron affinity and high absorptivity in the visible region. Fan et al. copolymerized DPP containing 2-octyldodecyl (OD) alkyl chain with BBT to obtain P-BBT-7 which exhibits a broadened absorption peak in 1,333 nm (Zhang et al., 2019). SPNs of P-BBT-7 modified with Pluronic F-127 exhibit an *η* of 60% and exhibit a strong PA imaging signal at 1,280 nm to achieve the effect of passively targeting tumors in PA imaging of subcutaneous xenograft tumor-bearing mice (Zhang et al., 2019).

### TBZ-Based Near-Infrared Window-II Semiconducting Polymer Nanoparticles

Compared with the analog of BBT, the advantage of the (1,2,5)thiadiazolo(3,4-*f*)benzotriazole (TBZ) unit is that it provides an opportunity on the N atom of the triazole ring to incorporate a solubilizing alkyl chain while maintaining high electron-withdrawing capability (Dong et al., 2013). Liu et al. used the SPNs of P-TBZ-1 containing TBZ and DPP unit, which shows an *η* of 67% and mass extinction coefficient of 43 ml mg^−1^ cm^−1^, to image a glioma tumor with a depth of 3.8 mm in a mouse’s skull (Yang et al., 2019). Liu et al. designed SPNs of P-TBZ-2 as an exogenous contrast agent with a resolution of 19.2 µm and an SBR of 29.3 dB in microscopy imaging of mice ear, indicating that it can be potentially applied to assist 3D optical-resolution photoacoustic microscopy imaging in various biomedical applications (Guo et al., 2019). Zhen et al. constructed SPNs of P-TBZ-3 with ultrasmall size, which exhibited an *η* of 53% and specific targeting in a tumor-bearing nude mice model (Men et al., 2020).

### ATQ-Based Near-Infrared Window-II Semiconducting Polymer Nanoparticles

ATQ, as an analog of BBT, can be alkylated to improve the solubility of polymers with a stronger electron-accepting ability compared to TBZ (Perzon et al., 2007). Bian et al. copolymerized an acceptor unit, ATQ, with a donor unit, benzo(1,2-*b*:4,5-*b′*)dithiophene, to obtain P-ATQ-1 exhibiting an absorption peak at 929 nm and a vibronic shoulder at 1,030 nm (Wen et al., 2020). Under 1,064 nm excitation, SPNs of P-ATQ-1 generate a strong PA signal with a mass extinction coefficient of 13.25 cm^−1^ mg^−1^ ml, which can passively target tumor sites in a brain tumor model. Subsequently, Bian and his coworkers prepared a positively charged SPN consisting of a hydrophobic P-ATQ-2 core, an anionic interlayer, and a cationic shell (Yin et al., 2018). The PA signals generated by P-ATQ-2 can achieve a highly efficient PA labeling of stem cells, and the PA contrast increased by an amount of 40.6- and 21.7-fold in subcutaneous and brain imaging relative to unlabeled cases. Li et al. synthesized a series of (1,2,5)thiadiazolo(3,4-*g*)quinoxaline (TQ)-based SPNs, P-ATQ-3, P-ATQ-4, and P-ATQ-5, through substitution with functional groups to explore the molecular guideline for efficient non-radiative decay (Zha et al., 2020). Due to the strong electron-withdrawing capability of the ester-substituted TQ unit, P-ATQ-5 exhibits a larger dihedral angle, lower radiative decay, and narrower adiabatic energy than alkyloxyphenyl and alkylthienyl-substituted TQ SPNs. SPNs of P-ATQ-5 show an *η* of up to 60% and a signal increase of 26.44 and 22.35 times, respectively, in *in situ* subcutaneous and hepatic tumors, which maintained a clear PA tracking upon 20 days. Similarly, Liu et al. designed a series of ATQ-based SPNs (P-ATQ-6, P-ATQ-7, and P-ATQ-8) *via* a copolymerizing ester-substituted ATQ acceptor and various donor monomers, with peaking at 1,140 , 1,270, and 1,500 nm in P-ATQ-6, P-ATQ-7, and P-ATQ-8, respectively (Luo et al., 2021). Under 1,064 nm irradiation, SPNs of P-ATQ-7 enhanced the SNR by 10 times in a mouse cerebrovascular model with a tissue depth of 10 mm. Pu et al. designed P-ATQ-9 composed of ATQ as a acceptor unit and diketopyrrolopyrrole (DPP) as a donor exhibiting broadband absorbing from the NIR-I to NIR-II regions. Compared with the PA image at 750 nm, 1.5-times higher SNR can be obtained by using P-ATQ-9 at a depth of 3 cm PA images of brain vasculature under 1,064 nm irradiation, indicating the advantage of PA imaging within the NIR-II window (Jiang et al., 2017). P-ATQ-10 designed by Liu et al., composed of 6,6,12,12-tetrakis(4-hexylphenyl)-s-indacenodithieno[3,2-*b*]thiophene as an elongated π-system donor and ATQ as the acceptor, exhibits a mass extinction coefficient of 18 L g^−1^ cm^−1^ under 980 nm laser irradiation and a quantum yield of 1.25% in the NIR-II region, indicating that the NIR-II brightness is higher than that of most NIR-II SPNs (Yang et al., 2020). Bian et al. developed a novel SPN P-ATQ-11 fusing with hydrophilic PEG side chains in the ATQ segment, which is beneficial for improving the stability of SPNs *via* a self-assembly process of amphiphilic PEG side chains (Yin et al., 2020). Due to the broadband absorption of P-ATQ-11 in the NIR-II region, a higher SNR in chicken breast tissue imaging was achieved than that in PA imaging in NIR-I. This result shows that the flexible modification of the ATQ unit provides an effective molecular design methodology to improve the stability, brightness, and biocompatibility of SPNs.

## Conclusion

This review summarizes SPNs containing benzobisthiadiazole or its derivative segments for NIR-II PA imaging. Due to the high electron-withdrawing capability, BBT and its derivatives can be flexibly copolymerized with a variety of donors to obtain a number of SPNs in the NIR-II range with objectively excellent PA properties and *η*s. An ATQ or a BTZ unit with better solubility through incorporating solubilizing alkyl chains can be obtained by replacing one thiadiazole ring of BBT with triazole or quinoxaline rings while maintaining the electron-withdrawing capability close to BBT. By flexibly adjusting the side-chain properties of the ATQ unit, the twisted intramolecular charge transfer effect of the molecule can be effectively enhanced, thereby optimizing the photothermal conversion and photoacoustic performance for PA imaging in the mouse model. Current disease models for NIR-II PA imaging of BBT and its derivative-based SPNs are mainly superficial tumor models or brain tumor models. In order to achieve greater penetration depth in NIR-II PA imaging, novel SPs with higher possible absorption and extinction coefficients and photothermal conversion efficiency should be explored to offset the energy dissipation during the irradiation of deep tissues. Once the limitation of the detection depth makes a breakthrough, PA imaging based on SPNs will show great potential in the diagnosis of clinical cancers such as lung cancer and glioma due to its non-invasiveness and high efficiency. All in all, BBT and its homologs, as a strong electron-withdrawing acceptor with an easy-to-modify structure, exhibit a unique strategy for constructing efficient NIR-II photoacoustic agents.
